# A model to promote the uptake of male circumcision as an HIV-preventive measure in high HIV and low male circumcision prevalence settings

**DOI:** 10.4102/hsag.v24i0.1070

**Published:** 2019-02-11

**Authors:** Charles Maibvise, Thandisizwe R. Mavundla

**Affiliations:** 1 Department of Health Studies, University of South Africa, South Africa; 2 Department of General Nursing, University of Swaziland, Swaziland

## Abstract

**Background:**

Human immunodeficiency virus (HIV) and acquired immunodeficiency syndrome (AIDS) remain the leading global burden of disease, especially in Southern Africa. As such, efforts to develop innovative preventive and curative measures continue to be a global priority. Of late, the World Health Organization recognised and recommended mass male circumcision (MC) as an adjunct HIV-preventive measure in 14 selected sub-Sahara African countries. However, despite efforts to promote the uptake of MC in these countries, the uptake remains significantly below set targets.

**Aim:**

The purpose of this article is to describe the process that was followed in developing, describing and evaluating a model to promote the uptake of MC as an HIV-preventive measure in high HIV and low MC settings.

**Setting:**

The model is designed for all settings of high HIV and low MC prevalence.

**Method:**

A theory-generative, qualitative, exploratory, descriptive and contextual research design was used. The process involved four distinct steps, namely concept analysis, description of relationship statements, and description and evaluation of the model using the criteria of clarity, simplicity, generality, accessibility and importance.

**Results:**

The central concept was identified as ‘promote the uptake of MC’, and three integral constituents were identified for the process, such as transforming men’s mindsets about MC, facilitating accessibility and utilisation of MC services, and maintaining a supportive social system. These formed the basis for the model.

**Conclusion:**

The model provides a framework of reference for healthcare providers in promoting the uptake of MC as an HIV-preventive measure in high HIV and low MC settings.

## Introduction

Over the last 10 years, male circumcision (MC) gained much fame and popularity among traditionally non-circumcising groups. This followed the recommendation by World Health Organization (WHO) and the Joint United Nations Programme on HIV/AIDS (UNAIDS) that MC may be an adjunct human immunodeficiency virus (HIV)-preventive measure, particularly in the 14 selected Eastern and Southern African countries worst affected by HIV, but with the least MC prevalence (Grund 2010; WHO 2011a, 2011b). These are Rwanda, Uganda, Kenya, Ethiopia, Tanzania, Malawi, Mozambique, Zambia, Botswana, Namibia, Zimbabwe, South Africa, Swaziland and Lesotho (Grund 2010; WHO 2011a, 2011b). Mathematical modelling shows that attaining 80% MC coverage in the 14 selected countries would avert up to 3.36 million new infections by 2025, leading to a net saving of $16.51 billion (Njeuhmeli et al. 2011).

To date, the 14 selected Eastern and Southern African countries have considerably invested in human and material resources to scale up MC uptake (Bertrand et al. 2014). They hosted intensive MC awareness and demand generation campaigns for close to a decade. This resulted in nearly 11.7 million cumulative MCs being performed between 2008 and 2015. However, this is only 56% of the 80% MC global target set in 2011, to be attained by 2015. In addition, the UNAIDS Fast Track Programme has revised the MC target to 90% coverage by 2020, yet the rate of MC uptake continues to decline. A 19% fall in MC uptake was recorded between 2014 and 2015 (UNAIDS 2016; WHO 2016). Consequently, Sgaier et al. (2016) postulate that we need a systematic approach and framework to improve the uptake for MC. Although efforts are evidently being made to ensure that MC interventions are innovative and evidence-based (Evens et al. 2014; Semeere et al. 2016), there is no consolidated framework of reference for these interventions. As such, MC interventions are applied as discrete activities, as opposed to a comprehensive and complementary set of activities with a unified goal. Furthermore, the subjective understanding of the concept of MC among the target population continues to negatively affect acceptability of the procedure, hence the low uptake (Rennie et al. 2015).

The purpose of this study was to develop and describe a model that can be used as a framework of reference in promoting the uptake of MC as an HIV-preventive measure in high HIV and low MC settings – based on a conceptual meaning of ‘promote the uptake of MC’ as well as an integrated and holistic approach to MC interventions.

## Methodology

### Design

The researchers used a theory-generative, qualitative, exploratory, descriptive and contextual research design. The qualitative aspect of the design allowed for an in-depth and holistic investigation of the phenomenon of ‘promoting the uptake of MC’ through the collection of rich narrative materials using a flexible design (Polit & Beck 2012). This included a focus on the qualitative aspect of meaning and understanding of human experiences regarding MC from the perspective and context of people in high HIV and low MC settings (Brink 2010; LoBiondo-Wood & Haber 2010). The exploratory component of the design allowed a broad investigation of the nature of the phenomenon, including all the factors related to it. The intention was to shed more light on the various ways in which the phenomenon manifests and the underlying processes or mechanisms (Polit & Beck 2012). The descriptive aspect entailed an accurate account which clearly portrayed the characteristics of the phenomenon under investigation in real-life situations, with the intention to discover unknown human experiences regarding the phenomenon in their natural setting (Burns & Grove 2011; LoBiondo-Wood & Haber 2010). The contextual aspect implies the understanding and description of the phenomenon of promoting the uptake of MC within the specific context and boundaries of high HIV and low MC settings (Babbie & Mouton 2009).

As the name suggests, the overall goal of a theory-generative design is to develop a theory. A theory is a systematic abstraction of reality that serves some purpose. Theory development, therefore, literally entails discovering, describing and explaining the true and unbiased relationships between concepts that constitute a phenomenon of interest (Chinn & Kramer 2011). The goal of this study was to develop a model for promoting the uptake of MC. According to Chinn and Jacobs (1987), theories and models coexist. As a representation of reality, a theory requires some modelling for its development, and it may incorporate some models within it. A theory goes beyond the meaning of a model (Chinn & Jacobs 1987). As such, a model is often viewed as a precursor or foundation of a theory (Brink 2010). Models can therefore be a form or a level in the development of a theory. According to Polit and Beck (2012), models differ from theories mainly in that models are not formally tested, that is, they lack the deductive system of propositions that asserts and explains relationships between concepts. However, their purpose or function as well as the process of development remains the same, hence the suitability of the theory-generative design in this study.

### Method

The researchers followed the four steps of theory generation proposed in Chinn and Kramer (2011) in developing the model. These are concept analysis, construction of relationship statements, model description and evaluation of the model.

#### Step 1: Concept analysis

Walker and Avant’s (2011) eight-step method of concept analysis was used. The specific details on how the method was applied are elaborated in a separately published article (Maibvise & Mavundla 2017). This method was chosen mainly because of its widespread use in the nursing discipline (Walker & Avant 2011). The eight steps featured within the three main components or sub-steps of concept analysis, which are concept identification, concept definition and concept classification.

**Concept identification:** The central concept was derived from the researcher’s Master’s degree research study which explored men’s motives for undergoing MC (Maibvise 2012). The study involved collection of primary qualitative data through individual face-to-face interviews, followed by thematic analysis using Creswell’s (2009) six-step method of qualitative data analysis.

**Concept definition:** The central and associated concepts were defined based on an integrative review of related literature. The researchers conducted a rigorous open online search using the following key words: ‘promote’, ‘uptake’ and ‘male circumcision’. The researchers also considered subject-related hard copy literature from relevant stakeholders, and analysed the data qualitatively and thematically.

Concept classification: The defined concepts were then classified according to their relatedness, based on Dickoff, James and Wiedenbach’s (1968) survey list for concept classification which consists of agent, recipient (patient), context (framework), procedure, dynamics and terminus.

#### Step 2: Construction of relationship statements

The various categories of concepts were placed into relationships with each other and respective descriptive relationship statements were formulated. This formed a meaningful structure or tentative model for promoting the uptake of MC for HIV prevention, as described below.

#### Step 3: Description of the model

Chinn and Kramer’s (2011) guidelines for theory description were used to describe the following components of the model: the purpose of the model, assumptions on which the model is based, the context in which the model applies, the concepts which make up the model, theoretical definitions of the identified concepts, the relationships between or among concepts and the structure of the model. Subsequently, the researchers formulated the guidelines for the operationalisation of the model in the practice of healthcare practitioners. The model and its guidelines were evaluated through a critical reflection (Chinn & Jacobs 1987; Chinn & Kramer 2011).

#### Step 4: Evaluation of the model

The model was evaluated by expert practitioners who majored in community health nursing and public health in the nursing discipline. These were educationists with experience in model development. The criteria of clarity, simplicity, generality, accessibility and importance were used to guide the evaluation (Chinn & Jacobs 1987; Chinn & Kramer 2011). Based on the evaluation, the model was deemed compliant with the criteria and appropriate for the intended purpose.

### Trustworthiness

Guba’s (1981) model for assessing trustworthiness was used. Four components of trustworthiness were assessed: truth value, applicability, consistency and neutrality. These components were measured using the strategies of credibility, transferability, dependability and conformability, respectively (Guba 1981; Krefting 1991; Lincoln & Guba 1985). These strategies were applied during collection and analysis of data to derive the conceptual meaning of ‘promoting the uptake of MC’, as described in step 1. The same strategies were also applied by the model evaluators in step 4 of the research project.

### Ethical considerations

In obtaining and handling data for concept analysis, the researchers observed the four basic ethical principles in medical research, namely autonomy, beneficence, non-maleficence and justice (The Medical Research Council of South Africa 2003), throughout the study. Before conducting the study, the researchers sought ethical approval from the Health Studies Research and Ethics Committee of the University of South Africa (Ethical clearance certificate number HSHDC/338/2014) as well as from the National Health Research Review Board of the Swaziland Ministry of Health. The autonomy of individual informants during primary data collection was respected. During the integrative review of literature, permission from authorities of relevant institutions, or custodians of information was also obtained before data could be accessed or collected.

## Results

The following aspects of the resultant model will be described according to Chinn and Kramer (2011). This will include looking at the purpose, underlying assumptions, the context, theoretical definitions of concepts, relationship statements and the structure of the model.

### The purpose of the model

This model serves as a framework for healthcare providers to promote the uptake of MC, thereby reducing the rate of sexual transmission of HIV and moving towards an HIV-free generation. The model is structured based on the following assumptions.

### Assumptions on which the model is based

The assumptions on which this model is based are a synthesis of findings from the concept analysis rooted in the paradigmatic perspectives of Newman’s System Model (Neuman 1990), the Health Belief Model (Rosenstock 1974) and the naturalistic paradigm at large (Brink 2010; LoBiondo-Wood & Haber 2010). The assumptions are as follows:

- A health-related intervention is likely to be adopted if perceived benefits or the efficacy of the intervention outweigh perceived risks or barriers of adopting it.
- Men’s uptake of MC depends on their individual perception of the procedure, and these perceptions can be modified or influenced by knowledge levels, demographic factors and psychosocial factors, among other factors.
- The low uptake of MC is attributed to negative perceptions about the procedure, which are rooted in individual psycho-socio-cultural backgrounds or experiences, as well as misinformation and misconceptions about MC.
- Harmonising the understanding of the meaning of the uptake of MC in the context of HIV prevention helps to eliminate individual biases that may negatively influence men’s perceptions and decision to undergo MC.
- Individual knowledge and perceptions are dynamic. Thus, the specific misconceptions and negative perceptions that account for low MC uptake vary with time and from place to place.
- Men are in constant and mutually beneficial interaction with their environment (including the social environment) and dynamically shape each other to maintain their own stability as entities.
- Men’s environment can be modelled to provide a more positive stimulus known as cues to action, or become more enabling, conducive and/or promotive of the uptake of MC as a public health measure.
- Men are holistic and efforts to influence their behaviour should also be holistic. Thus, a multi-sectoral approach is necessary.
- The HIV-preventive effect of MC enables men to maintain some degree of system stability or optimum wellness, that is, freedom from HIV.
- There is no single HIV-preventive measure that is 100% effective and always feasible, hence the need for the methods to complement each other and include MC.

### The context in which the model applies

This model is applicable in all settings of high HIV and low MC prevalence, particularly in the 14 Eastern and Southern African countries identified by WHO as having a significant projected public health benefit to the mass MC strategy. These are Rwanda, Uganda, Kenya, Ethiopia, Tanzania, Malawi, Mozambique, Zambia, Botswana, Namibia, Zimbabwe, South Africa, Swaziland and Lesotho (Grund 2010; WHO 2011a, 2011b). The strategy is set to be engraved in the disease prevention component of formal healthcare systems of the respective countries, and an atmosphere of political commitment and multi-sectoral collaboration is crucial for its success.

In the following, we provide the definitions of the central and associated concepts of the model as they are applied to the context just described.

### Definitions of the central and associated concepts of the model

The central concept, ‘promote the uptake of MC’, was defined from the process of concept analysis as an advanced process in which a healthcare provider or professional actively advances the health of uncircumcised men by transforming their perceptions about MC through educative interactions, facilitating the availability and accessibility of MC service and maintaining a supportive social environment. This process leads to positive perceptions about MC, acceptance of MC, a supportive social system, perceived self-efficacy and uptake of MC.

In this context, MC refers to partial or complete surgical removal of the foreskin in men in a clinical setting by trained health professionals for various reasons.

The associated concepts were identified and defined in the context of this model as follows.

#### Trained health professionals (healthcare providers and/or practitioners)

These are people trained and certified by state-accredited training institutions to render specified healthcare services in the modern conventional health system (Information and Privacy Commissioner [Ontario] 2004). In this context, their scope of practice includes performing an MC procedure or assisting or participating in MC within a clinical setting. Healthcare providers and/or practitioners include doctors, nurses and counsellors, among others.

#### Uncircumcised sexually active men

This refers to all men aged 15–49 who are not circumcised.

#### Safe male circumcision

Male circumcision services rendered by trained health professionals within a clinical setting, using approved and accredited equipment while following the specific guidelines stipulated by WHO, to minimise the risk for complications (WHO 2009).

#### Clinical setting

A place designed, equipped and certified by relevant government authorities under the relevant statutes to provide healthcare services in the conventional modern healthcare delivery system. The scope of a clinical setting in this case should include the provision of safe MC services.

#### Service provision

The process by which healthcare providers make health services, specifically safe MC services, available and accessible to the population.

#### Individual perceptions

The way one thinks about, comprehends or understands someone or something (Jones and Bartlett Learning [Sa]; *Merriam-Webster Dictionary* 2018). Perceptions can be positive or negative, and are mainly influenced by knowledge.

#### Knowledge

This refers to familiarity, awareness or understanding gained through experience or study (*American Heritage^®^ Dictionary of the English Language* 2016).

#### Educative interactions

A mutual or reciprocal action intended to educate, enlighten, inform, illuminate, instruct and improve knowledge and/or understanding. It can be a learner–content interaction, learner–instructor interaction or learner–learner interaction (Moore 1989). In this model, instructor corresponds to healthcare provider, learner to uncircumcised men and content to information about MC. Their interactions can be mediated by the social system, in which case the social system can fulfil two roles: (1) the role of a learner when interacting with the healthcare provider, receiving health education, and (2) the role of an instructor when interacting with uncircumcised men, sharing the received health education. In any case, the interactions also serve to increase publicity and popularity of the procedure.

#### Publicity

The public attention gained through extensive media coverage, word of mouth or other means of communication (*WordReference Random House Unabridged Dictionary of American English* 2018). The intention of publicity is to make people know about something, in this case MC.

#### Popularity

The state or condition of being liked, admired, enjoyed, accepted, supported and done by many people (*Merriam-Webster Dictionary* 2018; *Oxford English Dictionary* 2018).

#### Health-seeking behaviour

A pattern of formal healthcare system utilisation among any group of people that involves using formal medical channels to restore or promote health (MacKian, [Sa]). Health-seeking behaviour in men can be cultivated by educative interactions with their social system or directly with healthcare providers, who then bear the responsibility to link them to healthcare.

#### Linking to care

The process of connecting men who seek or need MC services to MC service providers. This is done by directing service seekers to service providers and vice versa. One of the drives to seek the service is men’s perceived self-efficacy.

#### Perceived self-efficacy

This is the belief and confidence in one’s ability, competence or readiness to successfully execute a task, in this case undergoing MC. Like any other individual perception, it can be modified by several factors, including the social environment (Glanz, Rimer & Viswanath 2008; Jones and Bartlett Learning [Sa]; Stretcher & Rosenstock 1997).

#### Social support

Refers to ‘everyday behaviours that, whether directly or indirectly, communicate to an individual that she or he is valued and cared for by others’ (Ko, Wang & Xu 2013). It is a daily interactional or communicative process with the environment, and can be classified into informational and emotional support, among other types of classification. Social support is exhibited in a social system.

#### Social system

This is a patterned series of interrelationships existing between individuals, groups and institutions that form a coherent whole (*Merriam-Webster Dictionary* 2018). It can also be viewed as an interdependent set of cultural and structural elements that function as a unit (LeBlanc 2010). These elements include immediate family members, peers, friends, relatives and agents of socialisation such as formal educational institutions and religious entities. A *supportive social system* therefore refers to a social system which reinforces or brings about men’s desire to undergo MC.

The identified concepts were classified as follows.

### Classification of concepts

Based on Dickoff et al.’s (1968) survey list, concepts were classified as follows: agent, recipient (patient), context (framework), procedure, dynamics and terminus.

An agent is a person who takes an active role in producing a specific desired effect, in this case an increase in the uptake of MC. In the model, the agent is a healthcare provider, particularly a nurse practitioner who has majored in community health nursing or public health. Nurse practitioners view people from a holistic perspective.

A recipient is the person who receives something; in this case the recipient gets knowledge and MC services. The recipients in this study are all uncircumcised men aged 15 years and above. These men have a higher risk of contracting HIV and the autonomy to make their own informed decisions to or not to undergo the procedure.

Context refers to the setting, conditions or circumstances in which an event occurs. In this study, context refers to all settings of high HIV and low MC prevalence, including the 14 countries identified by WHO as targets for the mass MC strategy (Grund 2010; WHO 2011a, 2011b).

A procedure refers to the specific activities that need to be done to bring about the intended goal or terminus. In this study, procedure refers to all interventions and actions which are necessary to attain the desired state of high MC and low HIV prevalence. Broadly, these activities include influencing individual perceptions, facilitating access and utilisation of safe MC services and enhancing a supportive social system around men.

Dynamics are forces or properties which stimulate growth, development or change within a system or process. In this model, dynamics refers to the motives for undergoing MC. These include educative interactions which influence men’s perceptions about MC, availability of MC services and a supportive social system such as continued encouragements, also known as cues to action.

Terminus is the situation to be produced at the end of a process, and in this case is a state of high MC and low HIV prevalence.

Having identified and defined these concepts, it is equally important to take a closer look at their relationships.

### Formulation of relationship statements

Theories and models are basically a set of concepts related in specified manners. Relationship statements stipulate the ways in which the concepts are related in forming the substance of the theory (Chinn & Kramer 2011; Walker & Avant 2011). The following are the relationship statements proposed between and among the identified concepts:

- In order for the uptake of MC to increase, there should be positive perceptions among uncircumcised men, a provision of safe MC services, as well as a supportive social environment.
- Men’s perceptions about MC are influenced by their knowledge of the procedure.
- Knowledge is transmissible. It can be transmitted from healthcare providers to sexually active men.
- Men’s awareness of MC increases their tendency to seek the service.
- Clinical settings and trained health professionals are associated with safe MC services.
- An MC procedure is safer when performed in a clinical setting by healthcare providers.
- The primary role of healthcare providers is to create a positive perception of MC among men so as to increase the uptake of MC.
- Healthcare providers also ‘link to care’ men who are in need of the MC services.
- To promote the uptake of MC, multi-sectoral collaborative efforts are required.
- Social support is an important modifying factor to individual perceptions and hence the decision to be circumcised.

Chinn and Kramer (2011) note that relationship statements give rise to the structure of the theory, which is described in the next section.

### The structure and process of the model

**FIGURE 1:**
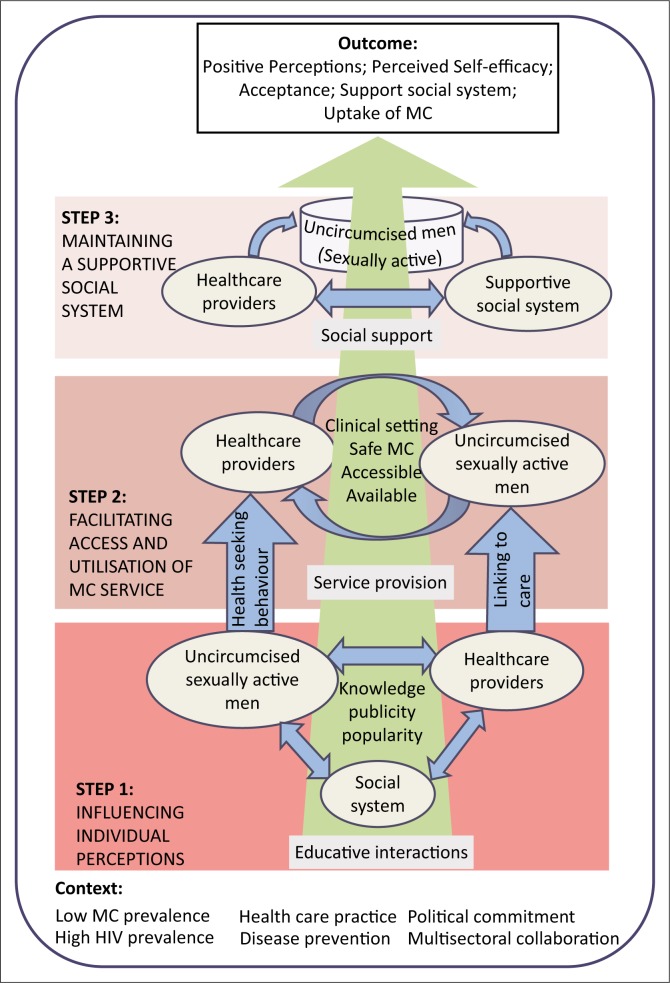
The model to promote the uptake of male circumcision.

A structure of a model refers to the overall form of the conceptual relationships within the theory (Chinn & Kramer 2011), while the process entails the steps that ought to be taken to attain the desired outcome. The two are intertwined, hence will be presented conjointly. Figure 1 illustrates the structure and process of the model.

The main features of the model include a green upward arrow which illustrates an overall transition from the current state of low MC and high HIV prevalence to the desired outcome of high MC and low HIV prevalence. The green colour symbolises growth and flourishing, and is associated with healing power from the rampant impact of HIV and AIDS (Color Wheel Pro, n.d.). The transition is driven by various interactions between healthcare providers and uncircumcised men, and is categorised into three building blocks, each corresponding to a specific step in the process as detailed below.

#### Step 1: Influencing individual perceptions

Individual perceptions are major determinants of behaviour change, and knowledge is an integral component of perceptions (Galloway 2003; Rosenstock 1974; Stretcher & Rosenstock 1997). This block consists of three reciprocal blue arrows signifying educative interactions among three elements: healthcare providers, uncircumcised sexually active men and the social system, in exchange of knowledge to generate publicity and popularity of MC among the men and their social system. Evidently, ignorance and misconceptions about MC are still prevalent among the target population (Hoffman et al. 2015; Rennie et al. 2015). It is envisaged that the enlightenment of men on MC will increase their health-seeking behaviour regarding MC services. Correspondingly, healthcare providers have an added responsibility to link these men to MC services. This demand generation exercise culminates in the need for step 2.

#### Step 2: Facilitating access to and utilisation of safe male circumcision services

The main role of healthcare providers at this stage is to facilitate availability, accessibility and utilisation of the MC services by men, as signified by the reciprocal blue arrows. Emphasis is placed on safe MC, which can only be availed in clinical settings. Notably, providing safe MC services is very expensive, ranging from about US$65.85 in Uganda to US$95.15 in South Africa (Njeuhmeli et al. 2011). Accordingly, a significant proportion of the needy population does not access HIV-preventive services such as MC (UNAIDS 2014, 2015).

#### Step 3: Maintaining a supportive social support system

In this final step, healthcare providers engage in collaborative efforts to support men’s social system to maintain the attained positive perceptions, acceptance and perceived self-efficacy. It has been noted that community leaders, sexual partners, peers and other relatives who constitute men’s social system greatly influence men’s decision to undergo circumcision (Galloway 2003). Religious beliefs as well as cultural norms and values are other influential components of the system. Together they form a class of modifying factors to individual perceptions known as cues to action (Galloway 2003).

The specific activities or guidelines that need to be followed to accomplish the just outlined three steps are described in the next section.

### Guidelines for operationalising the model

The guidelines were aligned to the procedure according to Dickoff et al.’s (1968) classification of concepts, which corresponds to the structure and process of the model. Thus, three distinct guidelines were formulated, each corresponding to a step in the model process. The guidelines were aimed at giving specific directions to healthcare providers in the targeted settings on how to influence uncircumcised men and the MC services delivery system to generate positive perceptions among men and increase the uptake of MC. Findings from the concept analysis process guided the formulation of the guidelines. In structuring these guidelines, the researchers observed the confinements and standards of UNAIDS and WHO’s joint strategic action framework to accelerate the scale-up of voluntary medical MC for HIV prevention in Eastern and Southern Africa (WHO 2009; WHO & UNAIDS 2011).

The feasibility and probable effectiveness of these guidelines, together with the entire model, were evaluated using the criteria described in the next section, as the final step in the model development process according to Chinn and Kramer (2011).

### Evaluation of the model

The expert healthcare professionals who evaluated the model concurred that the model complies with the criteria of clarity, simplicity, generality, accessibility and significance as proposed in Chinn and Kramer (2011). The concepts and the relationship statements were clearly defined, and the model structure clearly described. This was complemented by clear pictorial illustration of the models. The model was found simple because it was reduced to just three building blocks, consisting of only key concepts. The model was also considered sufficiently general, that is applicable to a wide range of situations, given that its purpose and scope were designed to apply to all settings of high HIV and low MC prevalence. Also, the model can be adapted for similar public health issues other than MC and HIV. The goals and outcomes of each key concept in this model, as well as those of the entire model, were found clear and attainable, with well-defined and observable indicators in the empirical world. The evaluation also pointed out numerous ways in which the model can potentially contribute significantly to clinical practice, education, administration and research regarding mass MC as an HIV-preventive measure. The next subsection captures some of the uniqueness contribution of the model.

### Unique contribution of the study

The model uniquely integrates all known key influential factors in the establishment and maintenance of the high uptake of MC. This integration occurs from a holistic perspective of men as the consumers of MC services. It factors in biological, economic, psychological, sociocultural and spiritual factors that influence MC uptake. This study has also produced a unique and refined conceptual definition for promoting the uptake of MC, which can be applicable across all settings of high HIV and low MC prevalence. The guidelines are also context-specific, thus taking into account some individual variations among communities in the selected study setting. Nonetheless, the researcher acknowledges the following limitations of the research process.

### Limitations of the study

The detailed concept analysis on which the model was developed is yet to be published. In addition, the practical implementation and validation of the model have not yet been done.

## Conclusion

The above limitations imply the possibility of further improving the model, probably as an ongoing process. The researchers intend to embark on that process in a series of postdoctoral studies. For the purpose of this study, whose overall objective was to develop and describe a model to promote the uptake of MC in high HIV and low MC prevalence settings, the researchers are confident that this objective was satisfactorily accomplished. More so because the researchers used empirically proven methodology, strictly adhered to and applied various critical methodological principles, and received a good evaluation outcome of the model from experts in model development. An accurate application of the proposed guidelines will yield the desired results.

Therefore, the researchers recommend that healthcare practitioners in different domains of the profession, namely clinical practice, education, administration, and research, start utilising the model in their professional practice. Broadly, for nursing or healthcare administrators, the model provides guidelines on how to ensure appropriate staffing and equitable distribution of safe MC services. The detailed guidelines will be published separately. Educators of health professionals can also get crucial insights necessary to enhance the professionals’ proficiency in the delivery of quality and safe MC services. To clinicians, the model serves as the empirical basis for different interventions in their evidence-based professional practice of mass MC for HIV prevention. The proposed relationship statements also serve as hypotheses for further research in the discipline of health. The researchers are certain that these contributions will collectively eventuate in the desired high MC and low HIV prevalent state.
